# The sickle cell trait and end stage renal disease in Salvador, Brazil

**DOI:** 10.1371/journal.pone.0209036

**Published:** 2018-12-17

**Authors:** Dona J. Alladagbin, Paula N. Fernandes, Maria B. Tavares, Jean T. Brito, Geraldo G. S. Oliveira, Luciano K. Silva, Nadia A. Khouri, Marilia B. Oliveira, Tatiana Amorim, Cácia M. Matos, Guilherme S. Ribeiro, Antônio A. Lopes, Marilda S. Gonçalves, Washington L. C. dos-Santos

**Affiliations:** 1 Instituto Gonçalo Moniz-FIOCRUZ-Bahia, Salvador, Brazil; 2 Universidade da Bahia, Salvador, Brazil; 3 Hospital Geral Roberto Santos, Salvador, Brazil; 4 Hospital Ana Nery, Salvador, Brazil; 5 Serviço de Referência em Triagem Neonatal—SRTN, Salvador, Brazil; 6 Instituto de Nefrologia e Diálise, Salvador, Brazil; University of Alabama at Birmingham, UNITED STATES

## Abstract

**Background:**

Carriers of the sickle cell trait (HbAS) usually remain asymptomatic. However, under conditions of low tissue oxygenation, red blood cell sickling and vascular obstruction may develop. Chronic kidney disease (CKD) can arise from conditions promoting low-oxygen in kidney tissue, which may be aggravated by the presence of the sickle cell trait. In addition, CKD can arise from other genetic traits.

**Aim:**

To compare the frequency of HbAS among hemodialysis patients and the general newborn population of Salvador (Bahia-Brazil), as well as to investigate the frequencies of apolipoprotein L1 risk variants in patients undergoing hemodialysis.

**Methods:**

A cross-sectional study included 306 patients with ESRD (End Stage Renal Disease) on hemodialysis for no more than three years. Hemoglobin profiles were characterized by high-performance liquid chromatography. To estimate the sickle cell trait frequency in the general population of Salvador, we analyzed data collected by a local neonatal screening program between 2011 and 2016. To exclude the potential contributing effect of the apolipoprotein L1 (*APOL1)* gene variants, we performed genotyping by PCR and DNA sequencing of 45 patients.

**Results:**

The frequency of HbAS was significantly higher in hemodialysis patients (9.8%) than in the general population (4.6%): Odds Ratio = 2.32 (95% CI = 1.59–3.38). No differences in demographic, clinical or laboratory data were found among patients with or without the sickle cell trait. The frequency of patients with none, one or two *APOL1* risk haplotypes (G1 and G2) for CKD were 80%, 18% and 2%, respectively.

**Conclusions:**

The frequency of the sickle cell trait is higher in patients with ESRD on hemodialysis compared to the general population. *APOL1* haplotypes do not seem to be the determinant of ESRD in these patients.

## Introduction

Sickle cell disease is a hereditary hemoglobinopathy caused by a glutamic acid replacement with valine in the hemoglobin β chain. This change leads to hemoglobin polymerization and erythrocyte sickling under conditions of low oxygen saturation. Sickled erythrocytes clump together, causing blood vessel obstruction that leads to tissue ischemia, resulting in acute chest syndrome, kidney papillary necrosis, stroke, spleen infarction and bone pain [1–4]. Heterozygote individuals with the sickle cell trait (HbAS) usually remain asymptomatic. However, they may rarely develop erythrocyte sickling, vascular occlusion and a disease that mimics what is observed in homozygote patients. For instance, spleen infarction and sudden death have been observed in heterozygote (HbAS) individuals under conditions favoring tissue hypoxia, acidosis or dehydration, such as in exhaustive physical activity performed at high altitudes, or below sea level [5–8]. The polymerization of hemoglobin S may lead to ischemia and infarction, increasing the risk of renal medullary carcinoma [9].

The sickle cell trait is frequent in the Brazilian population, varying from 1.1% to 9.8% [10]. In the state of Bahia, the region with the highest proportion of African descendants in the country, as well as many mixed-race individuals, the reported prevalence of HbAS varies from 5.3% in the general population to 9.8% among newborns at the Tsylla Balbino Maternity Hospital, which serves a predominantly black population in the capital city of Salvador [11].

Chronic kidney disease (CKD) is also frequently found in Brazil. In 2012, the estimated total number of patients on dialysis was 97,586. The estimated prevalence rates of dialysis were 503 patients per million and the annual gross mortality rate was 18.8% [12]. The main attributable causes of ESRD were hypertensive nephropathy, diabetic nephropathy and glomerulonephritis. All these kidney diseases evolve with vascular lesions that can lead to tissue hypoxia, e.g. both diabetes and hypertension induce atherosclerosis and hyaline arteriolosclerosis. Glomerulonephritis may evolve with endocapillary proliferation and capillary thrombosis, which impair post glomerular, including peritubular, circulation. Sickle cell disease has been associated with a variety of kidney lesions, including glomerular capillary sclerosis, infarcts and tubular dysfunction [13]. The vascular changes observed in sickle cell disease may more intensely affect the kidney medulla, which presents lower oxygen saturation and higher interstitial osmolality than other tissues. By contrast, the sickle cell trait is only infrequently associated with renal disease. Nonetheless, little is known regarding the contribution of HbAS to the severity and progression of inflammatory or degenerative kidney diseases, and the current literature is controversial with regard to this subject.

Naik and colleagues (2014) investigated alterations in kidney parameters in HbAS patients and found a higher prevalence of albuminuria (31.8% in HbAS patients vs. 19.6% in HbAA patients), lower glomerular filtration rates (22.6% in HbAS patients vs. 19% in HbAA patients) and a higher incidence of CKD (19.2% in HbAS patients vs. 13% in HbAA patients) [14]. These associations were found to be independent of other CKD risk factors. Derebail and colleagues (2010) also found a higher frequency of HbAS in African American individuals with CKD (15%) than in newborns (7%) in North Carolina (USA) [15]. However, similar studies performed in Rio de Janeiro and among African American individuals have shown no increase in HbAS frequency in patients on hemodialysis [16, 17].

When studying kidney disease in the populations of African descent, it is important to take into account other genetic factors also involved in progression of renal disease that may present a confounding effect. Certain variants in *APOL1*, a gene located on Chromosome 22 that encodes apolipoprotein L1 (APOL1), are associated with the progression of many kidney diseases [18]. The genetic variant G1 corresponds to a nucleotide mutation that results in an amino acid substitution at position 342 (S→G), which is associated or not with another that changes the amino acid at position 384 (I→M). The variant G2 is comprised of a six nucleotide deletion that leads to the absence of amino acids 388 and 389 in *APOL1* [19]. There is relatively little variance among prevalence rates of the CKD high-risk variants in the African-American population, in contrast to a high degree of variance among populations throughout Africa [20].

In this work, we compared the frequency of the sickle cell trait among patients with end-stage kidney disease to that observed in a historical series comprised of the general population of newborns in Salvador, Brazil. To assess the potential contribution of the *APOL1* gene to the progression to ESRD on hemodialysis patients with HbAS, we performed genotyping by PCR and used DNA sequencing to investigate gene variants in this group.

## Material and methods

### Ethics statement

This study was carried out in accordance with the recommendations of 466/2012 of the Brazilian National Health Council, and was approved by the Institutional Review Board for Research involving Human Subjects of the Gonçalo Moniz Institute, Fiocruz-BA, protocol number 382.273.

### Study design and patients

This cross-sectional study included all patients with ESRD on hemodialysis treatment for no more than three years between May 2014 and November 2015. We chose to exclude patients who had undergone hemodialysis for more than three years to avoid bias as a result of potential differences in survival between HbAS and HbAA patients on dialysis. The patients were seen at the following referral nephrology clinics and public hospitals in Salvador, Brazil: Ana Nery Hospital, Roberto Santos General Hospital and the Institute of Nephrology and Dialysis. Patients HbSS, HbSC hemoglobinopathy and thalassemia were excluded from the study. A total of 306 patients were recruited. To estimate the prevalence of HbAS in the general population of Salvador, data was obtained from the Bahia Neonatal Screening Program, performed from 2011 to 2016 by the Parents and Friends of Exceptional Children (APAE).

### Data collection and sample collection

Demographic, clinical and laboratorial data were collected from the patients’ medical records. Since skin color (self-reported or recorded by an independent observer) may correspond, at least in part, to the ethnicity of the population in Salvador, this parameter was used as a surrogate estimate of ethnic distribution in the group of patients with ESRD. For comparison purposes, self-referred data on skin color produced by the Brazilian Institute of Geography and Statistics (IBGE) was used to estimate the race distribution of the general population of Salvador. For each patient, a whole blood sample of 5 mL was collected by venopucture into ethylenediaminetetraacetic acid (EDTA)-containing glass tubes. Part of the samples were used immediately, and the remaining was stored frozen at -20 C until the time of use.

### Hemoglobin characterization

The patterns of hemoglobin distribution were measured by high-performance liquid chromatography. Hemoglobin was characterized using a VARIANT II β-Thalassemia Short Program Reorder Pack—Bio-Rad, USA), in accordance with supplier recommendations.

### DNA isolation, PCR and sequencing

A subgroup of 45 patients (25 HbAA and 20 HbAS) were selected randomly for molecular characterization of the DNA segment encoding part of *APOL1* serum-resistance-associated (SRA) domain. Human DNA was purified from the 200 μL blood samples using reagents and materials from the Qiagen DNeasy Blood and Tissue Kit following the manufacturer's instructions (Qiagen, Valencia, USA) and resuspended in 100 μL AE buffer. PCR were performed using oligonucleotide primers *APOL1* 6del seq F 5’-ACCAACTCACACGAGGCATT and *APOL1* 6del seq R 5’-CTGCCAGGCATATCTCTCCT, as previously described [21]. Briefly, PCR were carried out under the following conditions: 0.5 or 5 μL of purified DNA solutions, 0.2 μM of each primer, 0.2 mM of dNTP (Invitrogen) and 2 mM of MgSO4 with the use of Platinum Taq DNA Polymerase High Fidelity (Invitrogen). PCR products were treated with ExoSAP-IT Cleanup Reagent (ThemoFisher Scientific, USA) following manufacurer’s instructions. Sequencing in both directions were carried out using the inner primers 5’-CACGAGGCATTGGGAAGGACATC and 5’-AGGCATATCTCTCCTGGTGGCT on an ABI3100 automatic sequencer (Applied Biosystems, Foster City, USA).

### *APOL1* sequencing data analysis and genotyping

*APOL1* forward and reverse sequences were assembled to Genbank reference BC143038 using software CLC Main Workbench v.8.0 (Qiagen). Conflicting sites were confirmed by visual inspection of electropherograms. Three markers were analyzed: rs73885319, rs60910145 and rs71785313. Diallelic SNPs rs73885319 [A/G] and rs60910145 [T/G] were genotyped homozygous in the presence of a single peak, and heterozygous when a double peak was identified in both sequences. rs71785313 *insdel* [-/ATAATT/TTATAA] was genotyped homozygous or heterozygous when sequences in both directions aligned perfectly or aligned until the deletion site and thereafter showed overlapping nucleotides, respectively. Genotype of each patient by marker was recorded in an Excel spreadsheet and risk haplotypes were deduced whenever possible as G0 [A-T-*Ins*], G1^GM^ [G-G-*Ins*], G1^GI^ [G-T-*Ins*], and, G2 [A-T-*Del*] [22], and their frequencies were calculated.

### Statistical analysis

Statistical analyses were carried out using Prism software, version 5.01 (GraphPad, San Diego, CA, USA) and Stata IC software (StataCorp LP, College Station, TX, USA) version 11. Demographic, clinical and laboratory data were expressed as absolute numbers or percentages and were summarized as means and standard deviations where appropriate. Comparisons between HbAA and HbAS groups were performed using t-test or Mann-Whitney (non-parametric) test when applicable. Differences in proportions were analyzed using the chi-square test. Data on the frequency of haplotypes G0, G1 and G2 or the frequency of patients with 0, 1 or 2 *APOL1* risk alleles were expressed as the percentage of the total number of patients genotyped. Observed genotype frequencies were tested for Hardy-Weinberg equilibrium using GenAlEx 6.5 software [22]. Linkage disequilibrium (LD) was estimated by the r2 method and LD blocks were defined by the four gamete rule [23] using Haploview software [24]. Tests for statistical significance were considered when p < 0.05.

## Results

### Electrophoretic hemoglobin patterns

Data from the 1,055,614 newborns surveyed between 2011 and 2016 revealed that 4,181 (4.6%) had HbAS and 23,428 (2.2%) had HbAC (Table 1). Among the 306 hemodialysis patients recruited for hemoglobin analysis, 264 (86.3%) had HbAA, 30 (9.8%) had HbAS, and 12 (3.9%) had HbAC (Table 2). A statistically significant difference was observed between the prevalence of HbAS among patients subjected to hemodialysis and the general population Odds Ratio = 2.32 (95% CI = 1.59–3.38) p<0.0001. Furthermore, the prevalence of HbAC among patients subjected to hemodialysis was also higher than what was observed in the general population: Odds Ratio = 1.90 (95% CI = 1.06–3.40) p = 0.04.

**Table 1:** Frequency of hemoglobinopathies in the general population of newborns in Bahia-Brazil, from 2011 to 2016.

**YEAR**	TESTED	HbAS	(%)	HbAC	(%)	HbSS	(%)	HbCC	(%)
**2011**	187,234	8,490	(4.5)	4,254	(2.2)	110	(0.06)	36	(0.02)
**2012**	176,263	7,942	(4.5)	3,953	(2.2)	114	(0.06)	38	(0.02)
**2013**	173,303	8,022	(4.6)	3,733	(2.2)	119	(0.07)	45	(0.03)
**2014**	174,368	7,940	(4.6)	3,685	(2.1)	152	(0.09)	33	(0.02)
**2015**	177,185	8,058	(4.6)	3,939	(2.2)	125	(0.07)	41	(0.02)
**2016**	167,261	7,729	(4.6)	3,864	(2.3)	150	(0.09)	42	(0.03)
**TOTAL**	1,055,614	48,181	(4.6)	23,428	(2.2)	770	(0.07)	235	(0.02)

Source: Parents and Friends of Exceptional Children (APAE)

HbAS: Hemoglobin AS genotype; HbAC: Hemoglobin AC genotype; HbSS: Hemoglobin SS genotype; HbCC: Hemoglobin CC genotype

**Table 2:** Frequency of the hemoglobinopathies in patients with on hemodialysis in Salvador, Brazil.

HOSPITAL	TESTED	HbAS	(%)	HbAC	(%)	HbSS	(%)	HbCC	(%)
**INED**	103	07	(6.8)	02	(1.9)	00	(00)	00	(00)
**HAN**	84	10	(11.9)	07	(8.3)	00	(00)	00	(00)
**HGRS**	119	13	(10.9)	03	(2.5)	00	(00)	00	(00)
**TOTAL**	306	30	(9.8)	12	(3.9)	00	(00)	00	(00)

HbAS: Hemoglobin AS genotype; HbAC: Hemoglobin AC genotype; HbSS: Hemoglobin SS genotype; HbCC: Hemoglobin CC genotype. INED: Institute of Nephrology and Dialysis, HAN: Ana Nery Hospital, HGRS: Roberto Santos General Hospital

### Demographic, clinical and laboratory characteristics

No statistically significant differences were detected in most of the clinical or laboratory parameters evaluated, including iron status, iron dosage, erythropoietin dosage or blood transfusion among the patients with HbAA, HbAS or HbAC. A small difference in hemoglobin concentration was observed in HbAA patients (10.28 ± 1.9 g/dL) in comparison to HbAS (10.98 ±1.6 g/dL, Mann-Whitney test, p = 0.037). Nevertheless, blood hemoglobin concentrations in both groups remained below normal reference values, and despite this difference being statistically significant, there may well be no clinical relevance. Ferritin concentrations were highly variable in the three patient groups (variation coefficient of 88%, 80% and 61%). The frequencies of systemic arterial hypertension (approximately 90%) were high in three groups and diabetes (43%) in HbAA and HbAS (Table 3).

**Table 3:** Demographic, clinical and laboratory characteristics of patients on hemodialysis by hemoglobin genotype status.

PARAMETER							
	Number/Total or mean	(%) or SD	Number/Total or mean	(%) or SD		Number/Total or mean	(%) or SD
**N**	264/306	(86.3)	30/306		(9.8)	12/306	(3.9)
**Sex:**							
**Female**	112/264	(42.4)	15/30		(50.0)	6/12	(50.0)
**Male**	152/264	(57.6)	15/30		(50.0)	6/12	(50.0)
**Age**	52	15	56		14	55	10
**Ethnic group:**							
**Blacks**	54/214	(25.2)	12/28		(42.9)	4/10	(40.0)
**Mixed-race**	145/214	(67.8)	16/28		(57.1)	6/10	(60.0)
**Whites**	15/214	(7.0)	0/28		(0.0)	0/10	(0.0)
**Vintage/time on dialysis**							
**1 year**	167/195	(85.6)	20/195		(10.3)	8/195	(4.1)
**2 years**	48/56	(85.7)	5/56		(8.9)	3/56	(5.4)
**3 years**	48/55	(87.3)	5/55		(9.1)	2/55	(3.6)
**Cause of ESRD:**	112	(81.7)	23		(16.8)	2	(1.5)
**SAH**	39	(86.7)	05		(11.1)	1	(2.2)
**SAH and DM**	18	(78.3)	04		(17.4)	1	(4.3)
**DM**	17	(81.0)	04		(19.0)	-	-
**Glomerulopathy**	18	(90.0)	02		(10.0)	-	-
**PKD**	07	(87.5)	01		(12.5)	- 2.1x10^3^	-
**Obstructive uropathy**	01	(50.0)	01		(50.0)	-	-
**Undefined and others**	12	(66.7)	06		(33.3)	-	-
**Clinical and laboratory data:**							
**Current DM**	110/251	(43.8)	13/30		(43.3)	2/10	(20)
**Current SAH**	224/252	(88.9)	27/30		(90.0)	9/10	(90)
Hemoglobin (g/dL)**:	10.28	1.9	10.98		1.6	9.9	2.4
Hematocrit%*:	32.0	6.3	33.3		5.5	31.1	7.7
**Leucocytes(/mm**^**3**^**)x10**^3^**:	8.2 x10^3^	7.8 x10^3^	6.5x10^3^ 2.1x10^3^		2.1 x10^3^	7.6 x10^3^	3.5x10^3^
Albumin (g/dL)**:	3.7	0.6	3.7		0.5	3.7	0.5
Creatinine (mg/dL)*:	9.4	3.3	9.9		4.0	10.1	3.6
Urea (mg/dL)*:	146.3	45.0	149.5		49.0	148.0	39.1
Total cholesterol (mg/dL)**:	162.4	43.7	165.4		51.5	158.4	36.6
**Iron status:**							
Iron dosage (μg/dL)**:	63.0	32.4	67.9		26.6	64.6	13.8
Ferritin (ng/mL)**:	567.8	499.0	625.3		498.5	507.6	308.6
**Transferrin saturation %** **:	27.8	17.1	29.4		14.4	29.7	7.1
**Additional substitutive therapeutics:**							
**Blood transfusion**^**++**^:	16/220	(7.3)	2/28		(7.1)	1/9	(11.1)
**Erythropoietin dosage+:**	213/240	(88.7)	24/29		(82.8)	7/9	(77.8)
Erythropoietin dosage (mUI/ week)**:	10245	5405	10083		4548	11143	7105
**Iron dosage**^**+**^	146/240	(60.8)	18/29		(62.1)	5/9	(55.6)
**iron quantity (ampoule /** week)**:	1.7	0.9	1.7		0.8	1.4	0.9

* T test

** Mann-Whitney test; + Pearson chi2; ++ Fisher's exact. The values were expressed in frequency or mean ± standard deviation.

SAH: Systemic Arterial Hypertension; DM: Diabetes Mellitus; PKD: Polycystic Kidney Disease.

#### *APOL1* genotyping

**Fig 1:**
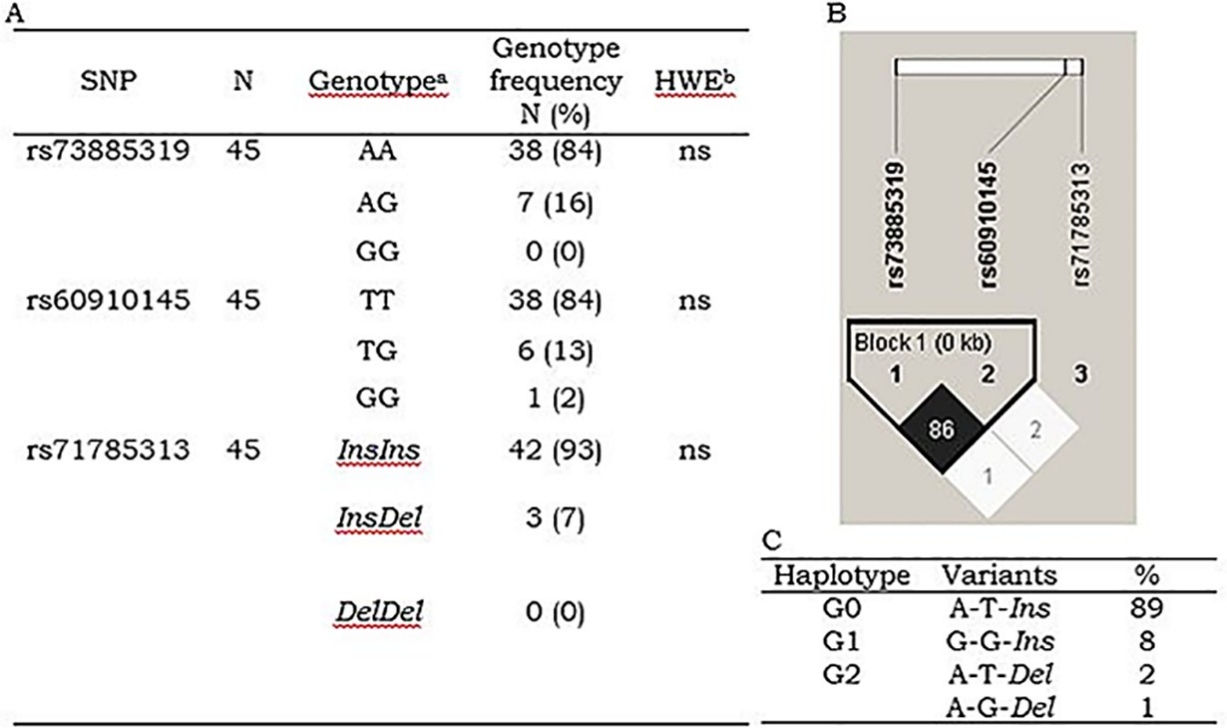
Genotype, allele, and haplotype frequencies of *APOL1* risk variants. (A) Genotype frequencies were. ns = not significant Chi-square goodness-of-fit test for Hardy-Weinberg equilibrium (HWE). (B) Linkage disequilibrium (LD) display. White bar represents relative distance. Numbers in the diamonds and color schema represents LD estimates (r2). Block 1 was defined by the four gamete rule. (C) Risk haplotype frequencies.

DNA samples of the 45 randomly selected patients were successfully sequenced and *APLO1* markers genotyped. All *APOL1* marker’s genotypes were in H-W equilibrium (Fig 1A). Markers rs73885319 and rs60910145 showed high LD, which supported the haplotype variants deduction (Fig 1B). Haplotype G0, G1 and G2 frequencies were 89%, 8% and 3%, respectively (Fig 1C). A new haplotype G2 [A-G-*Del*] variant was identified in one patient. The frequency of patients with 0, 1 and 2 risk haplotypes (G1 and G2) were 80%, 18% and 2%, respectively. No patient was homozygous for the risk haplotypes but one patient was compound heterozygous (G1/G2).

## Discussion

The present study found a frequency of HbAS almost twice as high in individuals with ESRD on hemodialysis (9.8%) than in the general newborn population (4.6%). This difference was similar to that reported in 2010 among African-Americans residing in North Carolina, USA [15]. In the same work, it was found an association between HbAS and decreased GFR and albuminuria [14]. Ajayi and colleagues (2010) also found a higher frequency of proteinuria in African patients with diabetes with the sickle cell trait compared to individuals with HbAA, and Peces and colleagues (2007) demonstrated that the sickle cell trait accelerates the progression of CKD arising from autosomal dominant polycystic kidney disease, a frequent cause of end-stage renal disease [25, 26]. These findings suggest that HbAS may constitute an independent risk factor for CKD development and/or progression to ESRD requiring hemodialysis.

In contrast, other studies in the literature have shown that HbAS is not a factor independently associated with susceptibility to CKD. For instance, Isaias and colleagues (2013) and Hicks and colleagues (2012) found no differences when comparing the prevalence of HbAS among adult patients with ESRD on dialysis and the general population [16, 17], despite the fact that each study employed a different design. Hence, an alternative explanation for the observed difference in the prevalence could be the selection of comparison group that do not properly represent the population from where the cases originated. Indeed, we used a dataset of newborns screened for hemoglobinopathies between 2011 and 2016 as a surrogate of the frequency of HbAS in the general population. In the case that there was a decrease in HbAS prevalence in the population of Salvador, Brazil in the last decades, we might have overestimated the HbAS prevalence difference. It, however, seems unlikely that a substantial change of similar magnitude occurred in both studied populations in Salvador and North Carolina, which would then be reflected herein and in the report by Derebail and colleagues (2010) [15]. Furthermore, the frequency of HbAS has remained unchanged in Salvador since 2011 when screening for the sickle cell trait became widely implemented (Table 2). Another possibility is that the sickle cell trait would confer a survival advantage, as was proposed for countries where *P*. *falciparum* malaria is prevalent. However, no condition that favors patients with S hemoglobin has been identified in Brazil to date.

Two other possible explanations for the found increased prevalence of HbAS in patients with ESRD on hemodialysis may be socioeconomic determinants and associated gene polymorphisms favoring renal disease progression.

Despite recent improvement, social inequality remains high in Brazil, impairing access to proper health care, particularly among populations of African descent, in which the prevalence of HbAS is greater. Therefore, it is possible that HbAS prevalence actually represent a confounding variable for the association between low socioeconomic status and ESRD. Hence, an increase in the proportion of HbAS individuals undergoing dialysis would be a reflection of the social condition they face, and not causally associated with the prevalence of the sickle cell trait. In fact, the proportion of black/mixed-race subjects reported by our study patients (94%) was substantially higher than what was estimated by the Brazilian Institute of Geography and Statistics (IBGE) for the general population of Salvador (80%). Unfortunately, the data regarding skin color were not collected in 54 (50 HbAA, 2 HbAS and 2 HbAC) patients in the beginning of the study, but there was no apparent bias in relation to ethnicity, SCT, *APOL1* gene variants in this subset of patients. Future studies should reexamine the association between HbAS and renal failure requiring dialysis adjusting for social economic indicators. In fact, a limitation of the current work is lack of a genetic principal components analysis adjusted to the degree of African genetic inheritance and it will be addressed in future studies.

The frequency of individuals heterozygous for HbAC is significantly higher in patients on hemodialysis (3.9%) than in the general newborn population (2.2%). This difference was similar to what was reported in 2010 by Derebail among African-Americans, in which a frequency of 5% was observed in patients on hemodialysis compared to 2% in the general newborn population [15]. Being heterozygous for HbAC may have a similar effect to HbAS and may be a cofactor for progression to end stage renal disease.

Wild-type *APOL1* is primarily considered as the human serum trypanolytic factor, protecting humans against the African trypanosomiasis causative agent *Trypanosoma brucei brucei*. Two variants of *APOL1* termed G1 and G2 play a protective role against the African trypanosomes *Trypanosoma brucei rhodensiense and Trypanosoma brucei gambiense*, parasites that cause the most deadly form of sleep sickness in humans [27]. However, these *APOL1* variants are also risk factors for kidney diseases, increasing the risk of CKD and ESRD in individuals of African and African-American descent [28–30].

Several scientific reports suggest that predisposition to develop renal diseases conferred by the G1 and G2 alleles follows a recessive inheritance model, in which homozygotes (G1/G1 or G2/G2) or compound heterozygotes (G1/ G2) show odds ratios varying from 11 to 68 to progress to kidney disease [18, 26, 31]. Heterozygous individuals (G0/G1 or GO/G2) show a low or non-detectable predisposition to the development of renal diseases [18, 32, 33]. In the current study, the frequency of haplotypes G0, G1 and G2 found were 89%, 8% and 3%, respectively. And the frequency of patients with 0, 1 and 2 *APOL1* risk haplotypes (G1 and G2) were 80%, 18% and 2%, respectively. Only one out of 20 patients with HbAS had the *APOL1* risk haplotype G1/G2. Only one out of 20 patients with HbAS had the APOL1 risk haplotype G1/G2. Although this haplotype was exclusive to this group of patients, neither APOL1 genotype frequencies nor risk haplotype frequencies were found to be significantly associated with HbAS. This result concurs with that reported by Naik and colleagues (2014), who found no association between the variants of APOL1 and HbAS in the development of the CKD in African-Americans [14].

## Conclusion

1. Patients with ESRD on hemodialysis in Salvador, Bahia-Brazil have:
  - a higher frequency of HbAS and
  - a higher frequency of HbAC,
    than the general population.
2. This association between HbAS and ESRD seems to be independent of *APOL1* gene polymorphisms.

Additional studies assessing the pathophysiology of sickle cell trait and its impact on renal disease could may provide important information to further the development of strategies to prevent the progression of CKD to ESRD.
